# Disruption of Glutamate Homeostasis in the Brain of Rat Offspring Induced by Prenatal and Early Postnatal Exposure to Maternal High-Sugar Diet

**DOI:** 10.3390/nu14112184

**Published:** 2022-05-24

**Authors:** Jozef Mizera, Bartosz Pomierny, Anna Sadakierska-Chudy, Beata Bystrowska, Lucyna Pomierny-Chamiolo

**Affiliations:** 1 Department of Toxicology, Faculty of Pharmacy, Jagiellonian University Medical College, Medyczna 9, 30-688 Krakow, Poland; jozef.mizera@uj.edu.pl; 2 Department of Biochemical Toxicology, Jagiellonian University Medical College, Medyczna 9, 30-688 Krakow, Poland; bartosz.pomierny@uj.edu.pl (B.P.); beata.bystrowska@uj.edu.pl (B.B.); 3 Department of Genetics, Faculty of Medicine and Health Sciences, Andrzej Frycz Modrzewski Krakow University, Gustawa Herlinga-Grudzinskiego 1, 30-705 Krakow, Poland; asadakierska-chudy@afm.edu.pl

**Keywords:** maternal high-sugar diet, offspring, extracellular glutamate, medial prefrontal cortex, hippocampus, EAAT1, EAAT2, VGLUT1, x_c_^−^

## Abstract

A high-calorie diet has contributed greatly to the prevalence of overweight and obesity worldwide for decades. These conditions also affect pregnant women and have a negative impact on the health of both the woman and the fetus. Numerous studies indicate that an unbalanced maternal diet, rich in sugars and fats, can influence the in utero environment and, therefore, the future health of the child. It has also been shown that prenatal exposure to an unbalanced diet might permanently alter neurotransmission in offspring. In this study, using a rat model, we evaluated the effects of a maternal high-sugar diet on the level of extracellular glutamate and the expression of key transporters crucial for maintaining glutamate homeostasis in offspring. Glutamate concentration was assessed in extracellular fluid samples collected from the medial prefrontal cortex and hippocampus of male and female offspring. Analysis showed significantly increased glutamate levels in both brain structures analyzed, regardless of the sex of the offspring. These changes were accompanied by altered expression of the EAAT1, VGLUT1, and x_c_^−^ proteins in these brain structures. This animal study further confirms our previous findings that a maternal high-sugar diet has a detrimental effect on the glutamatergic system.

## 1. Introduction

Rates of obesity, including maternal obesity, increased dramatically in recent decades. Estimates indicate that being overweight affected approximately 38.9 million pregnant women in 2014 worldwide, and 14.6 million of these women could be classified as obese [1]. The main cause of obesity is a calorically dense diet, rich in fats and sugars. A significant increase in the consumption of sweetened beverages and baked goods containing sucrose and high-fructose corn syrup is particularly evident in recent decades [2,3]. This obesogenic nutrition style was associated with detrimental consequences, not only for the health of mother, but also for her future children.

In the literature, a concept linking prenatal and early postnatal exposure to environmental factors with long-term consequences for the health of offspring is called Developmental Origins of Health and Disease (DOHaD). These potentially adverse factors include maternal nutrition, stress, hormonal perturbations, and xenobiotics [4]. Recent studies indicate epigenetic changes in the developing organism as an underlying molecular mechanism of this phenomenon [5]. The concept of DOHaD is supported by numerous studies that demonstrate that maternal diet is a significant determinant of the health of the offspring.

It was confirmed that an unbalanced diet of a pregnant mother and/or maternal obesity is associated with negative outcomes for the health of her children. Epidemiological data show that children of obese mothers are at higher risk of metabolic diseases, e.g., coronary heart disease, stroke, type 2 diabetes, and asthma [6]. Maternal obesity was also linked to behavioral and neuropsychiatric disorders in children, including hyperactivity, anxiety, aggressive behavior [7], learning impairment [8], depression [9], and schizophrenia [10]. Animal studies provide further confirmation. In offspring of mice, a maternal high-fat diet impaired hippocampal neurogenesis [11,12] and decreased BDNF level [13]. Behavioral changes caused by a maternal high-fat diet can manifest themselves through impaired spatial learning (mouse offspring) [13] and increased anxiety-like behavior (rat offspring) [14]. Research on offspring of nonhuman primates showed that a mother’s high-fat diet induced anxiety-like behavior along with disturbed serotonergic transmission [15,16], as well as reduced central dopamine signaling [17].

The results of the studies mentioned above show that an imbalanced maternal diet can disturb neurotransmission and induce neuropsychiatric abnormalities in the offspring. It is also well known that learning deficits, anxiety, depression, and addiction are linked with disturbances of glutamate system [18,19]. Our previous study on the influence of maternal high-sugar diet (HSD) in rats revealed numerous changes in the expression of NMDA receptor (NMDAR) subunits in the offspring [20]. A significant reduction in expression was observed in adolescent offspring, while adult offspring showed opposite changes. Furthermore, levels of miRNAs that regulate the activity of NMDARs were disrupted and disturbances in learning and memory were observed—both adolescent and adult offspring of mothers fed HSD showed impaired spatial memory.

**Figure 1:**
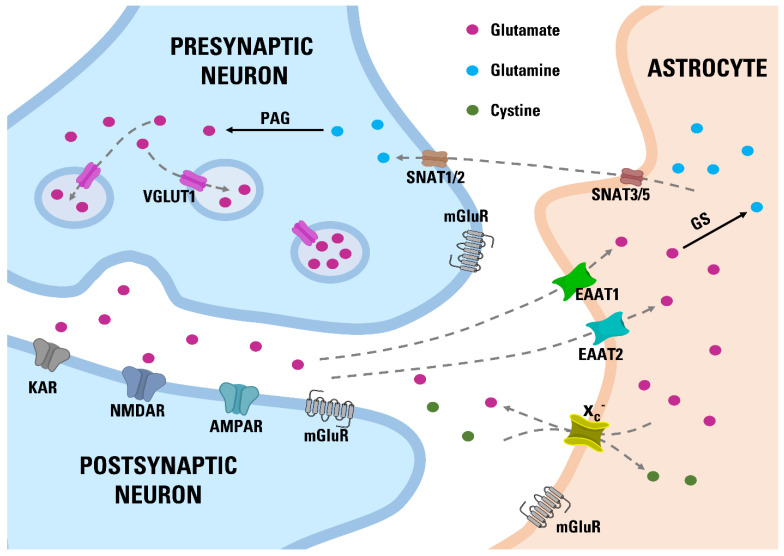
Glu—glutamine cycle in the central nervous system. PAG—phosphate activated glutaminase; GS—glutamine synthetase; KAR—kainate receptor; NMDAR—NMDA receptor; AMPAR—AMPA receptor. Glu released from the presynaptic neuron and that has not been bound to any receptor (NMDAR, AMPAR, KAR, mGluR) is collected by the astrocytic transporters EAAT1 and EAAT2. On the other hand, the antiporter x_c_^−^ releases Glu from astrocytes to extracellular space while importing cystine. Within the astrocyte, Glu is converted to glutamine by GS and then removed by the SNAT3 and SNAT5 transporters [31]. The released glutamine is taken into neurons by the SNAT3 and SNAT5 transporters, where the PAG converts it back to Glu. VGLUT1 then loads Glu into synaptic vesicles, where it awaits for the next release [32].

Glutamate (Glu) is the most abundant free amino acid and a major excitatory neurotransmitter in the human brain [21]. Glu is an agonist of ionotropic NMDARs, AMPA receptors, kainite receptors, and metabotropic receptors (Figure 1). Under physiological conditions, Glu is especially important for synaptic plasticity, learning and memory [22].

Due to the fact that released Glu is not degraded by any enzyme, it is, therefore, collected by specific transporters—EAATs (Figure 1). In rodents, EAAT1, EAAT2, and EAAT3 are referred to as GLAST, GLT-1, and EAAC, respectively. For simplicity, we will use only the EAAT term in this article. Each type of EAAT transports Glu into the cell; however, they differ greatly in their kinetics, localization, and abundance. EAAT1 and EAAT2 are the most abundant EAAT subtypes and are responsible for most of the Glu reuptake. Disturbances in EAAT expression have been associated with numerous neurological disorders, including epilepsy, Alzheimer’s disease, Huntington’s disease, multiple sclerosis, and amyotrophic lateral sclerosis. In most of these diseases, reduced levels of specific EAAT are observed with resulting increased oxidative stress and excitotoxic damage [23].

The proteins responsible for transferring Glu into the vesicles are vesicular Glu transporters (VGLUTs). The presence of VGLUT1 and VGLUT2 is characteristic for glutamatergic neurons, while VGLUT3 was identified in non-glutamatergic neurons [24]. Most Glu is transported to synaptic vesicles by VGLUT1 [25]. VGLUT1 and VGLUT2 usually show complementary and non-overlapping distributions in the brain. A large body of evidence indicates that VGLUT disturbances are associated with learning and memory deficits, as well as neurodegenerative disorders [26,27,28,29,30].

Cystine/Glu antiporter (x_c_^−^) is another element that modulates extracellular Glu concentration (Figure 1). This protein is expressed primarily in astrocytes [33]. x_c_^−^ is formed by the association of the light chain xCT and the heavy chain 4F2hc. Unlike EAATs, the x_c_^−^ system increases the extracellular Glu concentration. In the process of molecular exchange, one intracellular Glu molecule is exported, and one extracellular cystine molecule is imported into an astrocyte. It is estimated that the antiporter x_c_^−^ is responsible for more than half of non-synaptic Glu release [34]. Imported cystine is rapidly reduced to cysteine, which is used for glutathione synthesis [35]. It was established that x_c_^−^ activity is associated with numerous processes in the brain, e.g., anti-oxidative protection [36], modulation of neurotransmitter release [37], drug addiction [38], glioma growth [39], viral infection [40].

The purpose of this study was to evaluate changes in the Glu system caused in the offspring by maternal HSD. For this purpose, we used a rat model of prenatal and early postnatal exposure to a special diet enriched in sugars (sucrose, fructose, glucose). In the HIP and mPFC of the obtained offspring, we determined the levels of the extracellular Glu and the expression of key Glu transporters: EAAT1, EAAT2, VGLUT1 and x_c_^−^.

## 2. Materials and Methods

### 2.1. Animals

The Wistar rat females were purchased from the Faculty of Pharmacy of the Jagiellonian University Medical College, Poland. Rats were housed 2 per cage in a room with a 12-h light/dark cycle, under standard laboratory conditions. The research was carried out according to the European Union directive on the protection of animals used for scientific purposes (EU Directive 2010/63/EU). All procedures performed were approved by the Ethics Committee of the Institute of Pharmacology of the Polish Academy of Sciences, Krakow (approval number 95/2016).

### 2.2. Diets

The diets used in the study were purchased from the specialized manufacturer, Wytwornia Pasz ‘Morawski’, Poland. The dams of the control group received a standard control diet (SCD): total energy value: 3188 kcal/kg, 12% of fat calories, 24% proteins, 64% carbohydrates (polysaccharides 390 g/kg, disaccharides 47 g/kg). The dams of the experimental group received a special HSD: total energy value: 3775 kcal/kg, 64% calories from sucrose (251 g/kg), fructose (129 g/kg), glucose (129 g/kg), and polisachharides (133 g/kg), 24% protein, 12% fat.

### 2.3. Experimental Design

**Figure 2:**
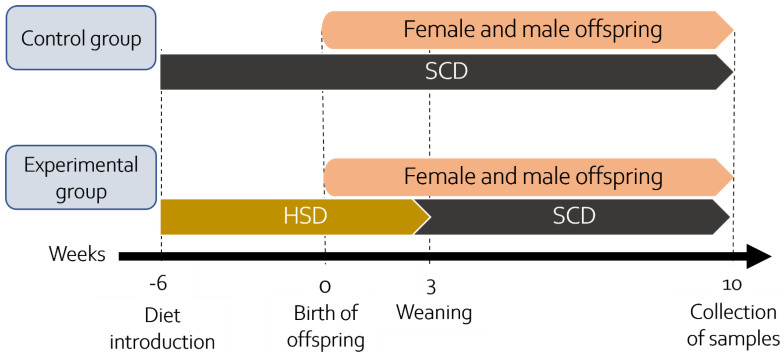
Graphic scheme of the experiment.

16 naïve rat females at 8 weeks of age weighing 190–220 g were randomly assigned to the control or experimental group. Dams from the control group were fed a SCD throughout the study. The dams of the experimental group received a special HSD for 9 weeks: 3 weeks before mating, pregnancy (3 weeks), and lactation (3 weeks). Feed and water were available ad libitum. The mothers had their glucose levels measured regularly; however, no significant differences were found. A few days before delivery, the pregnant dams were relocated to separate cages. Only offspring from litter size 8–12 with a similar number of females and males were used for further experiments (criteria established *a priori*). If the criteria were not met, the offspring were not included in research. Both mothers and pups were regularly weighed (see Figure S1 in Supplementary Data). The offspring were separated from the mothers on postnatal day 21 (PND21) and transferred to new cages according to sex and maternal diet. From PND21, the offspring of all groups were fed only SCD, and feed and water were available ad libitum. Four groups were obtained this way: SCD females (22 animals), HSD females (22 animals), SCD males (21 animals), and HSD males (21 animals). HSD females were compared to SCD females and HSD males with SCD males. Each animal was an experimental unit. In total, 86 offspring rats were used for experiments (30 for microdialysis, 32 for Western blot, and 24 for immunofluorescence assays) and grouped as follows: *n* = 7–8 per group for microdialysis, *n* = 6–8 for Western blot, *n* = 6 per group for immunohistochemical staining. The sample size meets the “reduction” criterion of the 3Rs principles, while providing reliable and statistically significant results. Tissues and samples were collected on PND70 during the light phase between 10 a.m. and 2 p.m. A graphic scheme of the experiment is presented in Figure 2. Rats at the age of 70 days are considered young adults [41]. In turn, in humans most cases of neuropsychiatric disorders, such as schizophrenia, depression, and bipolar disorder, manifest by early adulthood [42,43,44].

### 2.4. Tissue Sampling

Western blot analysis: the animal was decapitated and the brain was promptly removed. Brain structures: mPFC and HIP were dissected on an ice-cold glass plate according to the Paxinos and Watson atlas (coordinates: the mPFC—anteroposterior 3.72 mm to 2.52 mm from Bregma; the HIP—anteroposterior −1.72 mm to −6.84 mm from Bregma), then immediately frozen on dry ice and stored in a freezer at −80 °C until further analysis.

Immunohistochemical assays: the animal was injected with ketamine (80 mg/kg) and xylazine (20 mg/kg). The anesthetized animal was first transcardially perfused with phosphate buffer saline (PBS) and then with a 4% paraformaldehyde solution in 0.1 M PBS. The brain was then collected and placed overnight in the 4% paraformaldehyde solution at 5 °C. In the following days, the brains obtained were sequentially stored in 10%, 20%, and 30% glucose solutions. Finally, the brains were placed in a freezer at −80 °C.

### 2.5. Microdialysis

Animals had 24 h acclimatization period in the room adjacent to the laboratory where microdialysis was performed. The animal was anesthetized with 2.5% isoflurane and two guide cannulas (MAB 4, AgnTho’s, Lidingö, Sweden) were implanted in the stereotactic frame. One cannula was implemented in the mPFC (anteroposterior: +3.3 mm; mediolateral: +1.2 mm; dorsoventral: +5.0 mm) and another in the HIP (anteroposterior: 4.36 mm; mediolateral: +1.8 mm; dorsoventral: 2.5 mm). Using cranial screws and dental acrylic cement, the cannulas were fixed to the skull. The cannulas were plugged with a dedicated obturator. On the next day, artificial cerebrospinal fluid (aCSF) (NaCl 147 mM, KCl 4.0 mM, MgCl_2_ 1.0 mM, CaCl_2_ 2.2 mM, pH 7.4) was used to perfuse microdialysis probes (MAB 4, membrane with a molecular weight 6-kDa cutoff, 2-mm length, and 0.24-mm outer diameter, AgnTho’s AB, Sweden) at a 2 μL/min flow rate for 2 h. The probes were then inserted into the unplugged cannulas. The mPFC and HIP were first perfused with aCSF for 2 h. Samples were then collected for 1 h: the first 30 min to the first Eppendorf tube and next 30 min to the second tube. The tubes were placed in a tube stand on ice. The samples were immediately placed at −80 °C until further analysis. Each pair of samples was pooled before analysis. The investigator performing microdialysis was blinded to whether the rat was a control or experimental animal.

### 2.6. LC/MS/MS

A Shimadzu LC 20A HPLC system (Columbia, MD, USA) equipped with an auto-sampler, a degasser, and a Tandem Dual Plunger binary pump LC-20AT was used to determine the Glu concentrations. An Applied Biosystems/MDS Sciex (Concord, ON, Canada) API 3200 triple quadrupole mass spectrometer with an electrospray ionization (ESI) interface was used for MS/MS analysis. Chromatographic separation was performed with a ZIC^®^-HILIC (3.5µm, 100 Å) 150 × 2.1 mm (Merck, Darmstadt, Germany) in gradient mode. Analysis was performed according to Yan et al. (2019). [45] The column was thermostated at 40 °C and a mobile phase flow rate was set at 0.3 mL/min. The mobile phases consisted of 0.02 M formic acid in water (phase A) and 0.02 M formic acid in acetonitrile (phase B). The gradient began initially at 50% phase A for 1 min, increasing linearly to 95% for the next 2 min, then decreased to 50%. Finally, the last 4 min of analysis were kept at 50% to stabilize the baseline. The total analysis time was 7 min. The volume of the sample injected into the LC-MS/MS system was 6 µL. The sample temperature was maintained at 10 °C in the autosampler prior to analysis. ESI ionization was performed in the positive ionization mode. The multiple reaction monitoring (MRM) mode of the dominant product ion for Glu was used. The ion source parameters were as follows: ion spray voltage (IS): 5200 V; nebulizer gas (gas 1): 25 psi; turbo gas (gas 2): 20 psi; temperature of the heated nebulizer (TEM): 350 °C; and curtain gas (CUR): 25 psi. As the curtain and collision gas, nitrogen (99.9%) from Peak NM20ZA was used. Quantitation analysis was performed using the MRM mode. The ion pairs were monitored with values of m/z: 148.1/84.1 for Glu and 154.1/89.1 for Glu C13 (used as internal standard). The data obtained were analyzed using Analyst 1.6 software (Perlan Technologies). The concentration of the analyte was calculated using the standard calibration curve (Supplementary Data, Figure S2). The investigator performing the LC/MS/MS analysis was blinded to whether the sample belonged to the control or experimental group.

### 2.7. Western Blot

The mPFC and HIP samples were homogenized using a teflon-glass homogenizer in 200 μL sucrose buffer (0.32 M sucrose, 10 mM HEPES, 1 mM phenylmethylsulfonyl fluoride and 1 mM Na3VO4, protease and phosphatase inhibitor cocktail (Thermo Fischer Scientific, Waltham, MA, USA) pH = 7.4). Homogenates were first centrifugated at 1000× *g* and 4 °C for 10 min. The supernatants obtained were then centrifugated again (12,000× *g*, 20 min, 4 °C) to receive the membrane fraction in the pellet. This pellet was resuspended in 100 μL HEPES buffer (4 mM HEPES, 1 mM EDTA, pH = 7.4) and centrifuged (12,000× *g*, 20 min, 4 °C). The previous resuspension and centrifugation were repeated. The pellet was resuspended in 120 μL dissolving buffer (20 mM HEPES, 100 mM NaCl, 0.5% Triton, pH = 7.2) and then incubated for 15 min with gentle rotation at 4 °C. The samples were centrifuged (12,000× *g*, 20 min, 4 °C) and the obtained supernatant (non-PSD fraction) was collected and stored at −80 °C until further analysis.

Western blot analysis was performed according to Mizera et al. (2021). A BCA protein assay kit (Thermo Fischer Scientific) was used to normalize the protein concentration. The samples were then denatured with Laemmli Sample Buffer (Bio-Rad, Hercules, CA, USA) and loaded onto gels (stain-free gradient 4–15% SDS polyacrylamide gels, Bio-Rad) along with the protein marker (Spectra Multicolor Broad Range Protein Ladder, Thermo Fisher Scientific). After electrophoresis, proteins were transferred from the gels to the PVDF membranes. A Total Protein Staining kit (Thermo Fischer Scientific) was used to obtain staining of the entire membrane to normalize protein concentration and images were captured with a G:BOX Imaging System (Syngene). A representative image of PVDF membranes is included in the Supplementary Data (Figure S3). Staining was removed, the membranes were washed, then blocked and incubated with a primary antibody solution: anti EAAT1, anti EAAT2, anti VGLUT1, or anti xCT. In the next step, the membranes were incubated with secondary antibody and washed. Antibodies and their concentrations are listed in Table 1. Protein bands were visualized using Western Bright Quantum (Advansta) reagents and the G:BOX Imaging System. The signals obtained were matched with the respective signals of total protein staining. Due to the Western blot analysis requirement to know which samples are control or experimental, the investigator could not be blinded.

### 2.8. Immunofluorescence Assays

**Figure 3:**
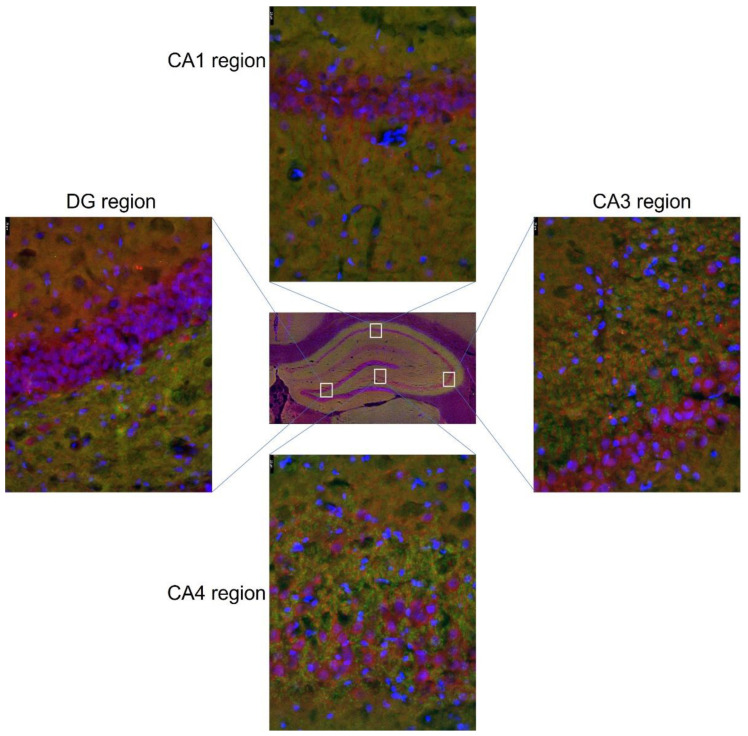
Hippocampal regions used in immunohistofluorescence analysis and their localization on the coronal section.

The perfused brains were cut into 20-μm coronal sections according to the Paxinos and Watson atlas (coordinates: the mPFC—anteroposterior 3.72 mm to 2.52 mm from Bregma; the HIP—anteroposterior −2.97 mm to −4.08 mm from Bregma) using a Leica CM1860 cryostat. The sections were placed on microscopic slides and stored at −20 °C until further analysis. The staining procedure was performed according to Mizera et al. (2021). The slides were washed in PBS, then in Triton X solution, and then in Tween20 solution. The sections were ringed with a hydrophobic marker; goat serum was applied and the slides were incubated. Next, primary antibodies (anti EAAT1 and anti GFAP, or anti EAAT2 with anti GFAP, or anti VGLUT1 and anti MAP2) were applied and slides were incubated. The list of antibodies used is presented in Table 1. The next day, the slides were washed and incubated with secondary antibodies. Due to the sensitivity to light of the secondary antibodies, the following steps were performed with minimal exposure to light. Slides were washed and VECTASHIELD Vibranc Antifade Mounting Medium with DAPI (VECTOR, Newark, CA, USA) was applied. The slides were covered with slip glass and sealed with clear nail polish. Images of the mPFC and HIP (CA1, CA3, CA4 regions, dentate gyrus (DG)) were obtained using a Leica DMI8 fluorescence inverted microscope with a Leica DFC450 CCD digital camera and Leica LAS X software. Sections were scanned in the *x*, *y* and *z* axes. The localization of the hippocampal regions is shown in Figure 3. Immunohistofluorescence assays were performed to evaluate signal intensity, visualize transporter localization, and also confirm Western blot analysis.

### 2.9. Statistical Analysis

The data obtained were analyzed using GraphPad Prism 8 software (San Diego, CA, USA). Normality of the distribution was assessed using the Shapiro–Wilk test. The 2-way ANOVA (diet × sex) multiple comparisons with the Benjamini and Yekutieli correction by controlling the False Discovery Rate was used to determine statistical significance. A *p* < 0.05 was considered significant. * *p* < 0.05; ** *p* ≤ 0.01; *** *p* ≤ 0.001. Results are presented as means ± SEM.

## 3. Results

### 3.1. Maternal HSD Increases Extracellular Glu Level in Both Female and Male Offspring

**Figure 4:**
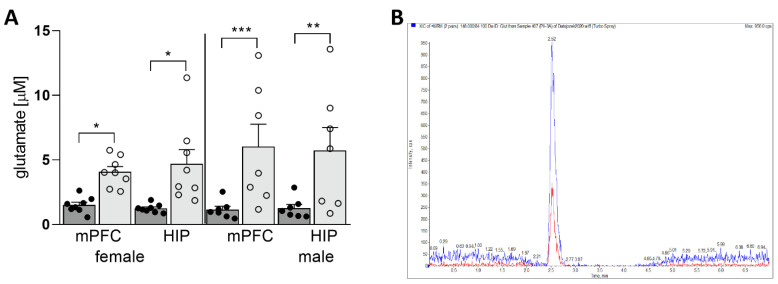
Concentration of extracellular Glu in the mPFC and HIP of the offspring (**A**). SCD—dark gray bars with black dots as individual data points, HSD—light gray bars with transparent dots as individual data points. Representative chromatogram (**B**). Blue peak—Glu; red peak—internal standard (Glu C13). * *p* < 0.05; ** *p* ≤ 0.01; *** *p* ≤ 0.001.

The LC/MS/MS method was used to assess the level of Glu in the microdialysis samples. A representative chromatogram is shown in Figure 4B. 2-way ANOVA analysis showed a significant influence of diet on Glu level in the mPFC (F_(1,26)_ = 19.08; *p* = 0.0002) and the HIP (F_(1,26)_ = 14.91; *p* = 0.0007). We found significantly increased Glu concentrations in each experimental group compared with its respective control (mPFC females: *p* = 0.0362; HIP females: *p* = 0.0209; mPFC males: *p* = 0.0006, HIP males: *p* = 0.0061) (Figure 4A). These results prompted us to test the expression of key transporters that maintain the proper level of Glu in the extrasynaptic space, such as EAAT1, EAAT2, VGLUT1, and x_c_^−^.

### 3.2. Expression of Glu Transporter Proteins (Western Blot)

**Figure 5:**
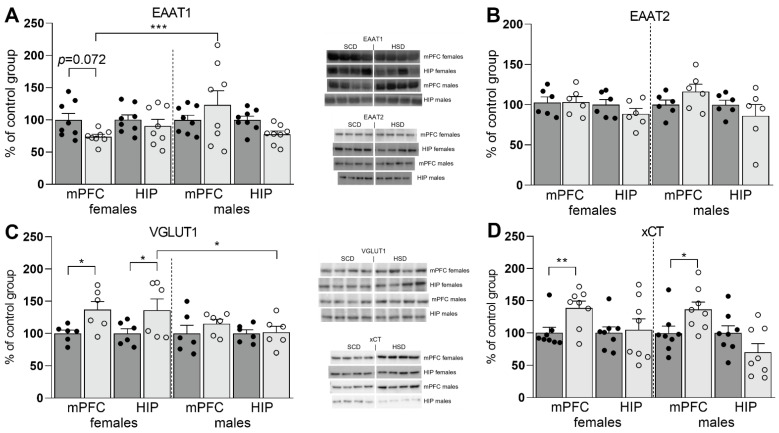
Expression of the EAAT1 (**A**), EAAT2 (**B**), VGLUT1 (**C**), and xCT (**D**) proteins in the mPFC and HIP of female and male off spring with representative immunoblots. SCD—dark gray bars with black dots as individual data points, HSD—light gray bars with transparent dots as individual data points.* *p* < 0.05; ** *p* ≤ 0.01; *** *p* ≤ 0.001.

Two-way ANOVA analysis showed that maternal HSD had a significant impact on the expression of the studied proteins in the mPFC (F_(7,96)_ = 3.93; *p* = 0.0008) and the HIP (F_(7,96)_ = 2.3; *p* = 0.0328). Moreover, in the HIP, the results are significantly influenced by sex (F_(1,96)_ = 4.29; *p* = 0.0411); however, no interactions between diet and sex have been demonstrated. The expression of EAAT1 was slightly reduced in the mPFC of female offspring (*p* = 0.072) prenatally exposed to maternal HSD. The direction of the changes in female and male offspring was opposite (*p* = 0.0008) (Figure 5A). The expression of EAAT1 in the HIP did not show significant changes. In turn, EAAT2 expression remained unaltered in both the mPFC and the HIP (Figure 5B). In the case of VGLUT1, an increase in expression was found in both the mPFC (*p* = 0.0286) and the HIP (*p* = 0.0174) of female offspring exposed to maternal HSD when compared with the control groups. No changes were found in the male group (Figure 5C). Also, the response of VGLUT1 expression to prenatal exposure to maternal HSD differed in males and females (*p* = 0.0236). A significant increase in the expression of the xCT protein was found in the mPFC of both female (*p* = 0.0085) and male offspring (*p* = 0.0121), while no changes were observed in the HIP (Figure 5D).

### 3.3. Expression of Glu Transporter Proteins (Immunofluorescence Staining)

**Figure 6:**
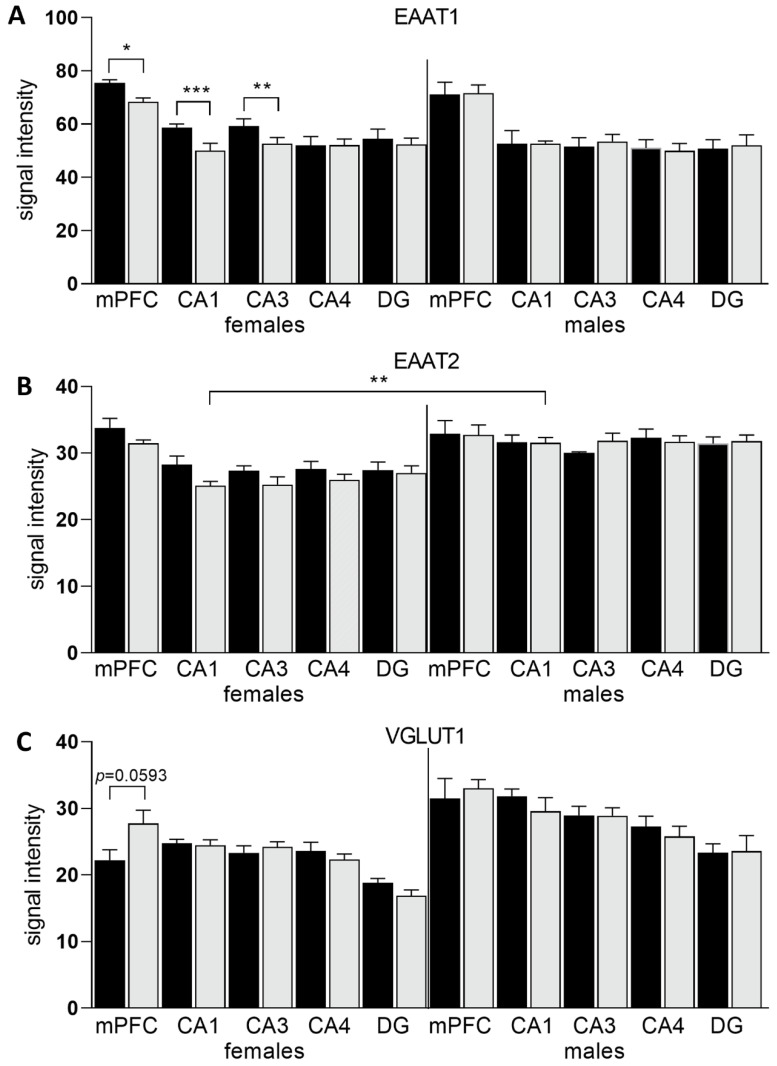
Intensity of immunohistofluorescence signals of EAAT1 (**A**) and EAAT2 (**B**) in astrocytes and intensity of the VGLUT1 (**C**) signal in neurons in the mPFC and hippocampal regions of female and male offspring. SCD—black bars; HSD—gray bars.* *p* < 0.05; ** *p* ≤ 0.01; *** *p* ≤ 0.001.

**Figure 7:**
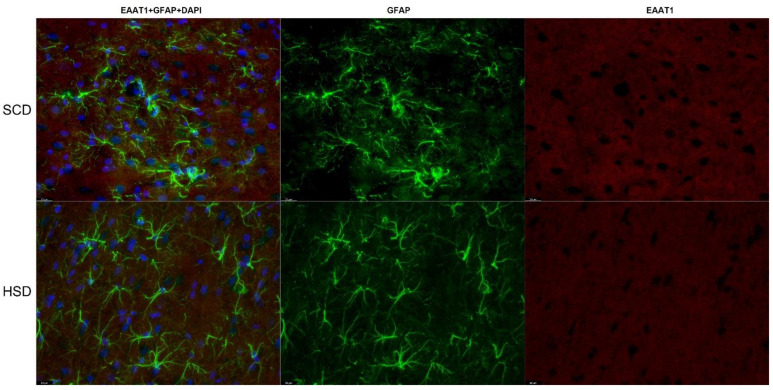
Immunohistofluorescence staining of EAAT1 localization on astrocytes in the mPFC of female offspring. Representative images showing EAAT1 immunoreactivity (red) in GFAP positive astrocytes (green) with DAPI-stained nuclei (blue). *n* = 6 in each group. Magnification ×40, scale bar 20 μm.

**Figure 8:**
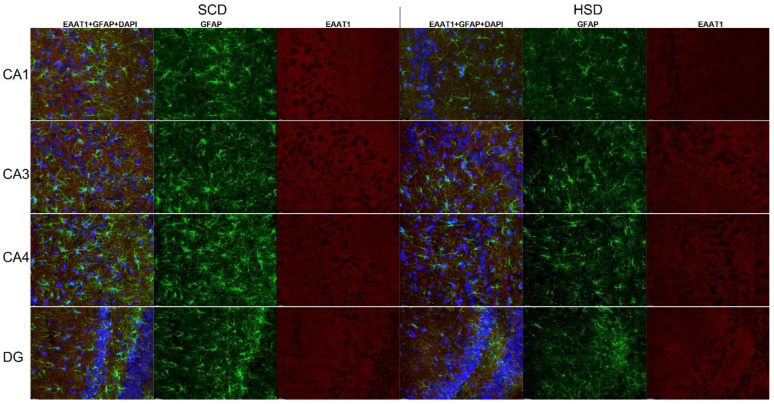
Immunohistofluorescence staining of EAAT1 localization on astrocytes in the HIP of female offspring. Representative images showing EAAT1 immunoreactivity (red) in GFAP positive astrocytes (green) with DAPI-stained nuclei (blue). *n* = 6 in each group. Magnification ×40, scale bar 20 μm.

**Figure 9:**
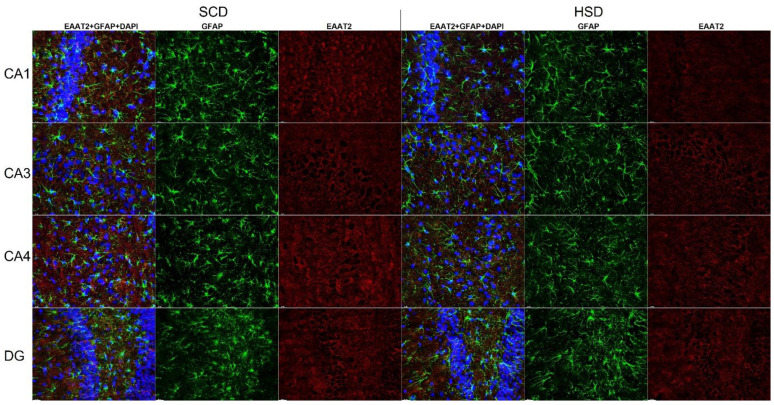
Immunohistofluorescence staining of EAAT2 localization on astrocytes in the HIP of female offspring. Representative images showing EAAT2 immunoreactivity (red) in GFAP positive astrocytes (green) with DAPI-stained nuclei (blue). *n* = 6 in each group. Magnification ×40, scale bar 20 μm.

**Figure 10:**
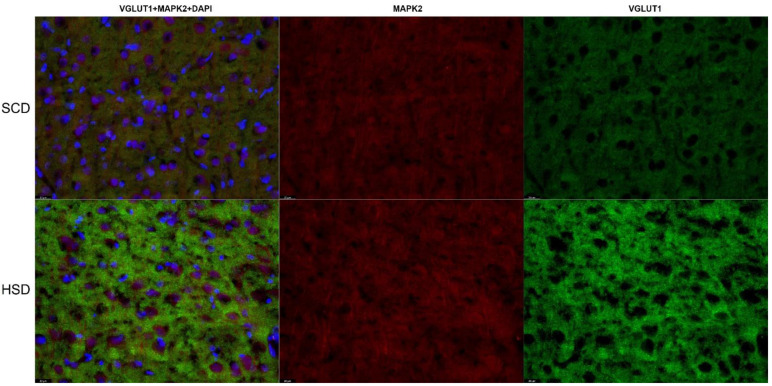
Immunohistofluorescence staining of the location of VGLUT1 in neurons in the mPFC of female offspring. Representative images showing VGLUT1 immunoreactivity (green) in MAP2 positive neurons (red) with DAPI-stained nuclei (blue). *n* = 6 in each group. Magnification ×40, scale bar 20 μm.

2-way ANOVA analysis revealed a significant influence of diet x sex interaction on the total variance in signal intensity of the studied proteins in both the mPFC (F_(5,60)_ = 2.439; *p* = 0.0459) and the HIP (CA1: F_(5,60)_ = 47.98; *p* < 0.0001; CA3: F_(5,60)_ = 47.10; *p* < 0.0001). Diet produced a significant effect on signal intensity of the studied proteins in the mPFC (F_(5,60)_ = 190.7; *p* < 0.0001) and the HIP (CA1: F_(5,60)_ = 47.45; *p* < 0.0001; CA3: F_(5,60)_ = 61.07; *p* < 0.0001). The intensity of the EAAT1 signal was significantly decreased only in female HSD exposed offspring in the CA1 (*p* = 0.0001) and CA3 (*p* = 0.0045) regions of the HIP, while in the mPFC a trend toward decreased values was observed (*p* = 0.0282). No changes were found in other hippocampal regions nor in male offspring (Figure 6A, Figure 7 and Figure 8). Immunofluorescence staining did not show significant differences in the EAAT2 signal in the structures analyzed in either female or male offspring when compared to their relative control groups; however, signal intensity in the CA1 region in HSD female offspring significantly differed from HSD male offspring (*p* = 0.0035) (Figure 6B and Figure 9). The VGLUT1 signals in the experimental group remained unchanged compared to the control. The results for the mPFC of males was close to statistical significance (*p* = 0.0593) (Figure 6C and Figure 10). Representative images of staining without significant changes are included in the Supplementary Data (Figures S4–S6). Immunohistofluorescence results for x_c_^−^ are not presented due to the lack of specific antibodies dedicated to this method. The commercially available antibodies we tested proved unsatisfactory specificity and largely bonded to neurons. A similar problem was reported in the literature [35,46,47].

## 4. Discussion

Our previous study revealed behavioral (sex-related memory impairment) and molecular (NMDA receptors hypofunction in brain structures) abnormalities in adolescent offspring exposed to maternal HSD during gestation and lactation, with normalization of most changes in young adult offspring [20]. In this study, we examined whether Glu homeostasis normalized in adulthood and whether there are any sex differences. We examined the basal extrasynaptic Glu level in brain structures associated with memory and cognition (mPFC and HIP) and evaluated the expression of proteins responsible for maintaining Glu homeostasis in the central nervous system.

The results of the present study indicate significant changes in glutamatergic system homeostasis. Analysis of the basal levels of extracellular Glu in free-moving animals showed a significantly higher concentration in the experimental groups of both females and males compared with control animals. The concentration of Glu in the control groups did not differ from each other. Interestingly, it seems that among the offspring there is a sex-dependent response to maternal HSD. In most of the female offspring, Glu levels were rather consistently elevated and low variability was observed. In turn, in male offspring there was greater individual variability. Some HSD male offspring showed a nondeviant or slightly increased Glu level compared with controls, while in other males Glu levels were extremely elevated. Explanation on molecular level of observed sex-dependent differences of Glu concentration requires further research. Combining these results with the results of our previous behavioral studies, where 70-day-old females showed impaired spatial memory [20], this indicates a greater predisposition of female offspring to develop a disrupted phenotype. It should be noted that in some of the observed cases of increased Glu level, its concentration reached the excitotoxic range (7–10 μM according to Hinzman et al. (2016) [48], while the physiological level in mammals is 0.2–3 μM [49]). Nevertheless, it seems that even if it is not an excitotoxic concentration, it may be enough to cause mild cognitive impairment. Increased glutamatergic activity in PFC with concomitant changes in the expression of genes of glutamatergic system receptors was also demonstrated in animals older than those used in our experiment (21–23 weeks) [50]; these changes, therefore, seem to be permanent. Hascup et al. (2019) described the effect of a high-fat diet on an elevated Glu level in the hippocampal subregions, while our results extend the risk of occurrence of such changes also to prenatal exposure to maternal HSD.

Like Hascup et al. (2019) [51], we observed a statistically significant increase in the expression of the vesicular transporter VGLUT1 in the female group, especially in the mPFC. On the other hand, mice with halved VGLUT1 expression exhibited normal spontaneous locomotor activity, increased anxiety in the light-dark exploration test, and depression-like behavior in the forced swim test, but showed no increased anxiety in the elevated plus maze test [30]. Additionally, in the novel object recognition test, the animals demonstrated normal short-term, but impaired long-term, memory with no changes in spatial memory assessed in the Morris water maze (*ibid*.). This suggests that presynaptic changes in glutamatergic synapses can lead to a behavioral phenotype similar to mental and cognitive disorders. Down-regulation of VGLUT1 was also found in Alzheimer’s disease animal models characterized phenotypically by memory impairment [52]. On the contrary, elevated levels of VGLUT1 were observed in a mouse model of Alzheimer’s disease with a high-fat diet [51]. Most studies describe down-regulation of VGLUT1 as an important factor in the degradation of the Glu system in many diseases, such as Alzheimer’s and Parkinson’s disease, and depression [25]. If so, why then is the expression of VGLUT1 increased in females in the HSD group in our study? We suspect that this may be the effect of developmental delay, because in the process of brain maturation, an increase in VGLUT1 expression is initially observed (in rats the peak falls at around 30 PND) followed by a gradual decrease [53]. In our research on NMDA receptors in this model, we observed a delayed switching of the ratio between the NR2B and NR2A subunits [20]; however, more research is required to confirm this hypothesis. It should be emphasized that significant changes in the expression of the VGLUT1 transporter were observed only in females. We also consider the possibility that elevated levels of VGLUT1 expression may be associated with mild gestational diabetes or insulin resistance [51]. The serum glucose level in the females during pregnancy was measured and did not differ from the control group; our model, therefore, does not qualify as a gestational diabetes model; however, the body mass of the female offspring was significantly reduced (figures in: Mizera et al., 2021), similar to the model of gestational diabetes [54].

No sex-related changes were found in the expression of the xCT protein of the x_c_^−^ anti-porter. Their significantly increased expression occurred in the mPFC in the offspring of both sexes. The x_c_^−^ transporter is not only an important regulator of extrasynaptic Glu levels [35], but is also an essential link in the antioxidant system [55]. Its expression and activity are intensified by oxidative stress and neuroinflammation. The system x_c_^−^ is regulated at the transcription level, and two transcription factors play a predominant role in the regulation of xCT expression—nuclear factor erythroid-derived 2-like (Nrf2) and activating transcription factor 4 (ATF4). Induced by oxidative stress and other stimuli, Nrf2 binds to the xCT promoter region. Nrf2 has been documented to regulate the activity of the x_c_^−^ system in astrocytes. It was found that not only a diet high in fat and fructose induces oxidative stress through the transcription factor Nrf2 [56], but dietary prenatal factors, such as high-fat exposure, can also interfere with this regulatory pathway [57]. ATF4 plays an important role in the resistance of neuronal cells to oxidative stress through direct transcriptional up-regulation of xCT [35]. Increased activation of this factor has been described in the hypothalamus of offspring exposed during pregnancy to a maternal high-fat diet [58]. Liu et al. (2020) [59] reported that high-fructose maternal diet induces neuroinflammation in adult offspring. Determining whether x_c_^−^ overexpression is beneficial or harmful in this model is not easy because of the involvement of this system in the processes of excitotoxicity and protection against oxidative stress. In contrast to intensified neurotoxicity due to Glu release, increased expression of the xCT protein may exert a protective effect through the production and release of glutathione. Increased oxidative stress in offspring from mothers fed an unbalanced diet was found in both preclinical [60,61] and clinical studies [62,63], hence overexpression of xCT appears to be a rather favorable adaptive response. Further research is required to evaluate the release of Glu from activated microglia in our model. Preclinical and clinical studies strongly suggest that excess fat and/or sugar in the mother’s diet also causes oxidative stress and inflammation in the nervous system in both the mother and the offspring [64,65]. Hence, the increased level of Glu in the mPFC and HIP is likely to be the result of a pro-oxidative effect of the diet.

This paper is the first report in the literature on the influence of the maternal diet on the expression of EAAT(s). Our results for the Western blot analysis did not show significant differences in the expression of the major extrasynaptic sweep transporter EAAT2 in either the mPFC or the HIP. EAAT2 signal intensity in the immunohistofluorescence analysis, however, was slightly reduced in HSD females in the CA1 field of the HIP in comparison with the control group, while compared with the HSD male group, significant reduction was observed. The CA1 hippocampal region is a critical brain area in spatial memory [66]. The significantly lower preference index in the new object recognition test in female offspring exposed to maternal HSD in our previous research [20] goes hand in hand with decreased expression of both EAAT1 and EAAT2 in this brain structure. Similarly, electrophysiological studies have shown that a high-calorie diet leading to obesity causes astrocyte hypertrophy, increased glutamatergic signaling, and impaired clearance via reduced EAAT2 function [67]. Reduction of EAAT1 expression observed in our study allows one to assume that the previously considered theory of insulin resistance could still be possible, as it is suggested that insulin-dependent down-regulation of EAAT1 is involved in the adjustment of Glu concentrations in synapses [68]; furthermore, EAAT1-mediated Glu transport is more efficient in Müller glial cells in diabetic patients [69]. Further studies would, therefore, be useful to assess the presence of silent changes in glucose metabolism despite the lack of evidence for the diagnosis of diabetes in test animals. Our results suggest that there is a risk of such changes in offspring at prenatal and early postnatal ages and that these could last at least until adulthood.

It should also be noted that in the experimental groups of our study there was a similar percentage of offspring with changes in the expression of transporters involved in the regulation of the Glu level (VGLUT1, EAAT2, or x_c_^−^). Because it was not possible to use the same animals for the analysis of both the Glu concentration and the protein expression, we are unable to estimate the correlation between the results of these two analyses.

In summary, our study is the first to present significant changes in Glu homeostasis in the mPFC and HIP of young adult offspring, after prenatal and early postnatal exposure to maternal HSD. The abnormalities discovered included an increase in extrasynaptic Glu concentration, VGLUT1 up-regulation, EAAT1 down-regulation, and xCT overexpression. It should be noted that some effects of maternal HSD on offspring seem to be sex-dependent, therefore future experiments with similar animal models should include both female and male offspring. Abnormalities observed in protein expression may explain molecular changes in the Glu system, as well as the cognitive disorders described in our previous paper, and may be associated with increased oxidative stress and abnormalities in glucose turnover; however, further studies are needed to verify these ideas.

## Figures and Tables

**Table 1:** List of antibodies used and their dilutions.

Target	Dilution	Manufacturer	Cat. Number
Western blot—primary antibodies			
EAAT1	1:1000	Abcam (Cambridge, UK)	ab416
EAAT2	1:500	Abcam (Cambridge, UK)	ab178401
xCT	1:1000	Abcam (Cambridge, UK)	ab175186
VGLUT1	1:1000	Abcam (Cambridge, UK)	ab77822
Western blot—secondary antibodies			
Goat anti-rabbit IgG	1:5000	Invitrogen (Waltham, MA, USA)	A27036
Immunofluorescence assays—primary antibodies			
EAAT1	1:300	Abcam (Cambridge, UK)	ab416
EAAT2	1:600	Abcam (Cambridge, UK)	ab41621
VGLUT1	1:1000	Merck Sigma Aldrich (Darmstadt, Germany)	AMAb91041
GFAP	1:1000	Abcam (Cambridge, UK)	ab4674
MAP2	1:1000	Abcam (Cambridge, UK)	ab5392
Immunofluorescence assays—secondary antibodies			
Goat anti-chicken AF488	1:300	Abcam (Cambridge, UK)	ab150173
Goat anti-rabbit AF594	1:300	Abcam (Cambridge, UK)	ab150080
Goat anti-chicken AF594	1:400	Thermo Fischer Scientific (Waltham, MA, USA)	A32759
Goat anti-mouse AF488	1:400	Thermo Fischer Scientific (Waltham, MA, USA)	A32723

## Data Availability

The original contributions generated for this study are included in the article; further inquiries can be directed to the corresponding author.

## Supplementary Materials

The following supporting information can be downloaded at: https://www.mdpi.com/article/10.3390/nu14112184/s1, Figure S1: Body mass of dams and offspring; Figure S2: Standard calibration curve for glutamate; Figure S3: Image of PVDF membrane after total protein staining (TPS); Figure S4: Immunohistofluorescence staining of EAAT1 localization on astrocytes in the HIP of male offspring; Figure S5: Immunohistofluorescence staining of EAAT2 localization on astrocytes in the mPFC of female offspring; Figure S6: Immunohistofluorescence staining of VGLUT1 localization on neurons in the HIP of the female offspring.
