# Supplementary material for: Disruption of Glutamate Homeostasis in the Brain of Rat Offspring Induced by Prenatal and Early Postnatal Exposure to Maternal High-Sugar Diet
Source: Nutrients. 2022 May 24;14(11):2184. doi: 10.3390/nu14112184 (PMC9182612; doi:10.3390/nu14112184)
Supplement: Supplementary file 1 [file nutrients-14-02184-s001.zip › nutrients-1684535-supplementary.pdf]

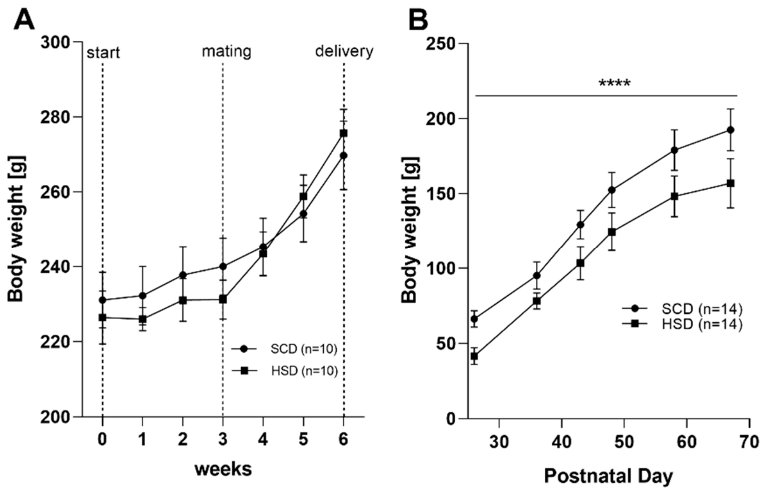

Figure S1. Body mass of dams before and during pregnancy (A). Body mass of female (B) and male (C) offspring from postnatal day 28 to 70.

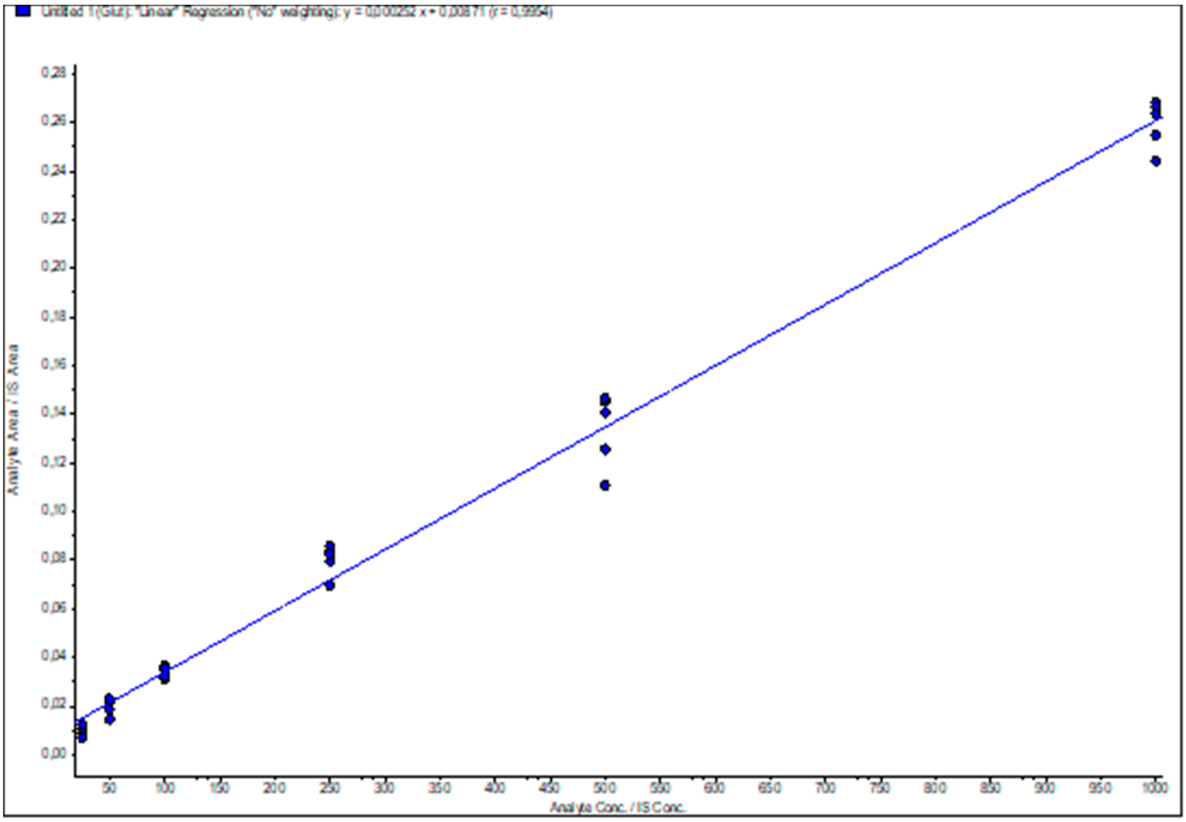

Figure S2. Standard calibration curve for glutamate used in LC/MS/MS analysis. Concentration in ng/ml.

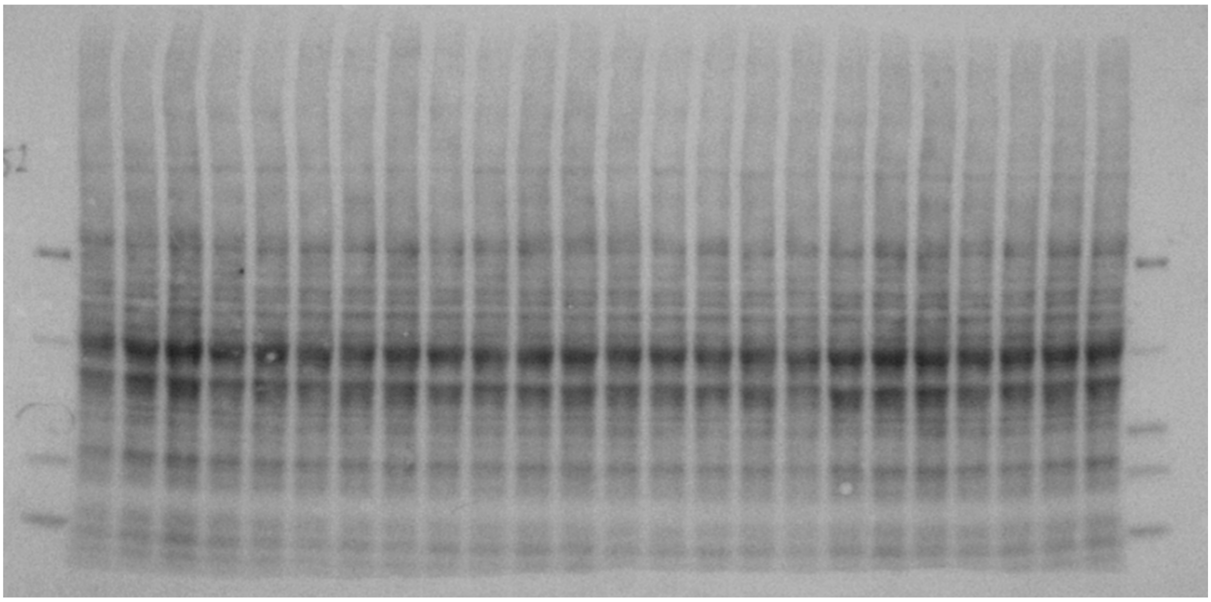

Figure S3. Image of PVDF membrane after total protein staining (TPS).

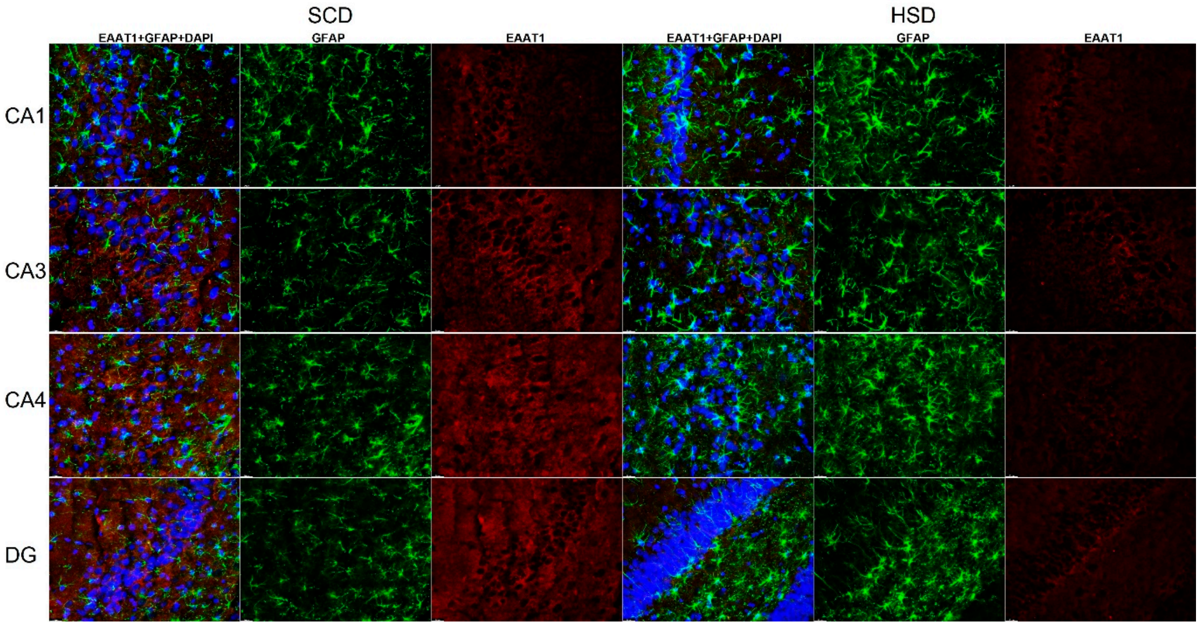

Figure S4. Immunohistofluorescence staining of EAAT1 localization on astrocytes in the HIP of male offspring. Representative images showing EAAT1 immunoreactivity (red) in GFAP-positive astrocytes (green) with DAPI-stained nuclei (blue). n = 6 in each group. Magnification × 40, scale bar 20 μm.

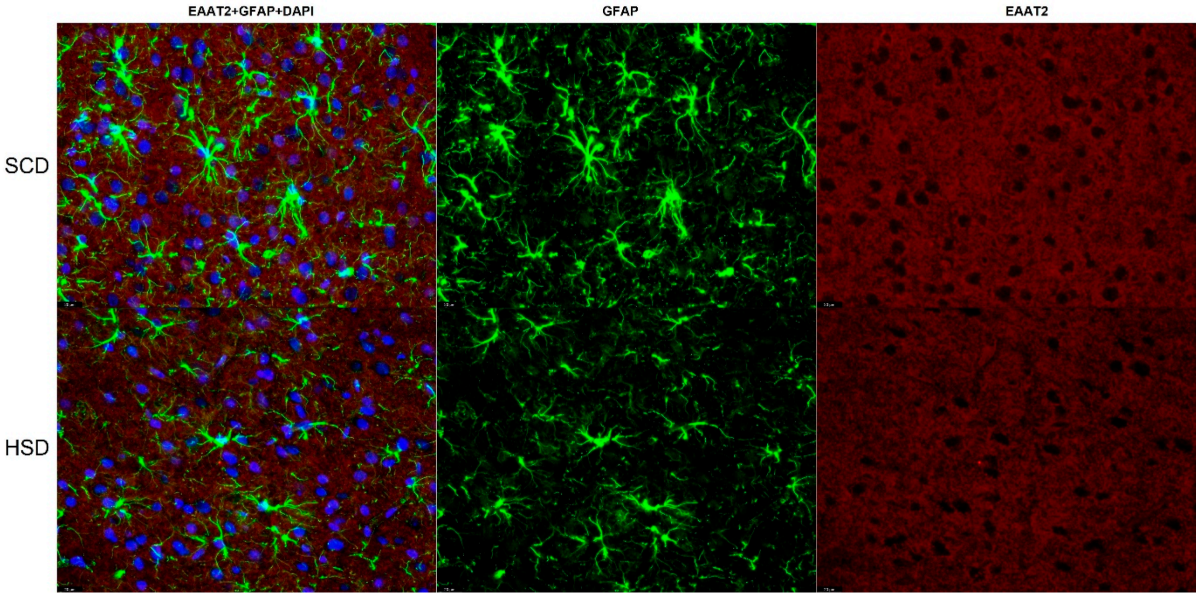

Figure S5. Immunohistofluorescence staining of EAAT2 localization on astrocytes in the mPFC of female offspring. Representative images showing EAAT2 immunoreactivity (red) in GFAP-positive astrocytes (green) with DAPI-stained nuclei (blue). n = 6 in each group. Magnification × 40, scale bar 20 μm.

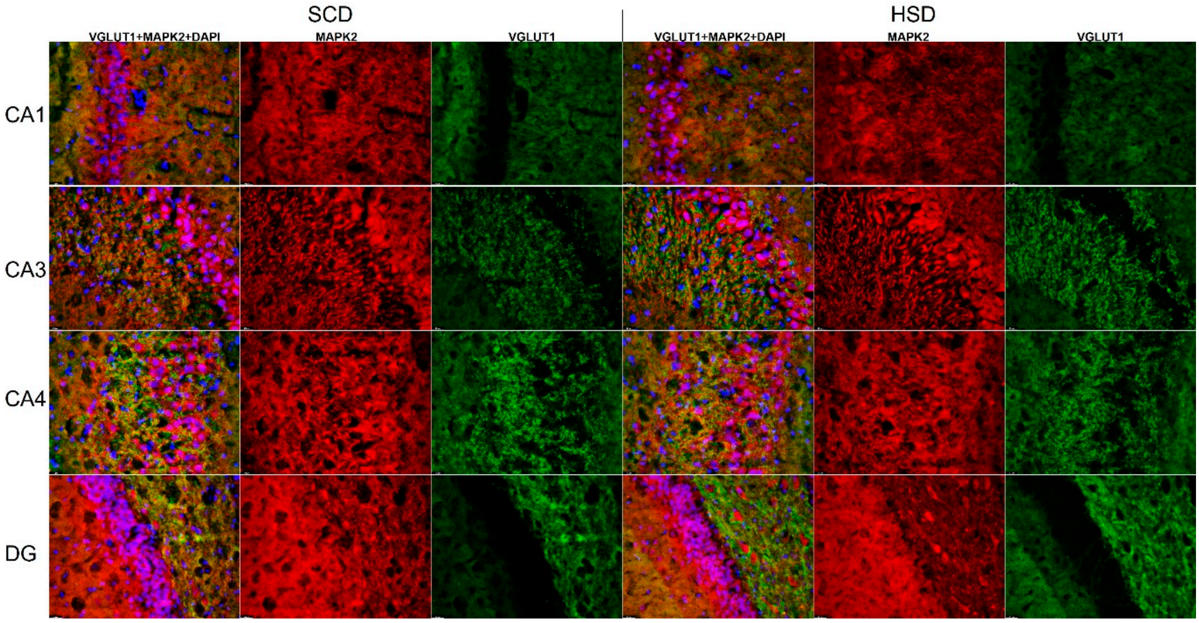

Figure S6. Immunohistofluorescence staining of VGLUT1 localization on neurons in the HIP of the female offspring. Representative images showing VGLUT1 immunoreactivity (green) in MAPK2-positive neurons (red) with DAPI-stained nuclei (blue). n = 6 in each group. Magnification × 40, scale bar 20 μm.
